# A multi-component classifier for nonalcoholic fatty liver disease (NAFLD) based on genomic, proteomic, and phenomic data domains

**DOI:** 10.1038/srep43238

**Published:** 2017-03-07

**Authors:** G. Craig Wood, Xin Chu, George Argyropoulos, Peter Benotti, David Rolston, Tooraj Mirshahi, Anthony Petrick, John Gabrielson, David J. Carey, Johanna K. DiStefano, Christopher D. Still, Glenn S. Gerhard

**Affiliations:** 1 Geisinger Obesity Research Institute, Danville, PA, USA; 2 Lewis Katz School of Medicine at Temple University, Philadelphia, PA, USA; 3 National Jewish Health, Denver, CO, USA.

## Abstract

Non-alcoholic fatty liver disease (NAFLD) represents a spectrum of conditions that include steatohepatitis and fibrosis that are thought to emanate from hepatic steatosis. Few robust biomarkers or diagnostic tests have been developed for hepatic steatosis in the setting of obesity. We have developed a multi-component classifier for hepatic steatosis comprised of phenotypic, genomic, and proteomic variables using data from 576 adults with extreme obesity who underwent bariatric surgery and intra-operative liver biopsy. Using a 443 patient training set, protein biomarker discovery was performed using the highly multiplexed SOMAscan^®^ proteomic assay, a set of 19 clinical variables, and the steatosis predisposing PNPLA3 rs738409 single nucleotide polymorphism genotype status. The most stable markers were selected using a stability selection algorithm with a L_1_-regularized logistic regression kernel and were then fitted with logistic regression models to classify steatosis, that were then tested against a 133 sample blinded verification set. The highest area under the ROC curve (AUC) for steatosis of PNPLA3 rs738409 genotype, 8 proteins, or 19 phenotypic variables was 0.913, whereas the final classifier that included variables from all three domains had an AUC of 0.935. These data indicate that multi-domain modeling has better predictive power than comprehensive analysis of variables from a single domain.

Obesity is associated with fat accumulation in the liver, which is commonly diagnosed as non-alcoholic fatty liver disease (NAFLD). NAFLD encompasses a wide range of conditions that are thought to arise from fatty liver (hepatic steatosis) to nonalcoholic steatohepatitis (NASH), which refers to findings on liver biopsy reflecting steatohepatitis (fat related inflammation) with or without fibrosis in the absence of significant alcohol consumption^1^,^2^. NAFLD has become the major cause of chronic liver disease due to the progression of simple steatosis to hepatocyte injury, liver inflammation, fibrosis, and cirrhosis that worsen clinical outcomes^3^,^4^ and are associated with increased liver-related morbidity and mortality^5^. The prevalence of NAFLD is increasing in tandem with the rising rates of obesity, and is expected to double in the U.S. by 2030^6^ with upwards of 100 million people in the U.S. at risk^7^. Despite the public health significance of NAFLD, most affected individuals remain undiagnosed^8^.

We^9^ and others^10^,^11^ have identified several clinical factors, including lipid and glucose levels, as well as parameters of iron metabolism, that are associated with the development of steatosis. However, neither clinical characteristics nor laboratory values have yet proved useful for predicting disease development or course^12^,^13^,^14^. Liver biopsy is the primary method used to accurately assess NAFLD stage^15^,^16^,^17^, although histological evaluation has diagnostic limitations^18^. Percutaneous liver biopsy is an invasive and expensive procedure associated with major complications, including mortality^19^. While liver biopsy can be performed in the context of abdominal surgery, particularly in patients with extreme obesity who are high risk for NAFLD undergoing bariatric procedures^18^, this population represents only a small fraction of patients at risk.

Given the substantial public health burden of NAFLD, robust methods are needed to identify those who have fatty liver who are at risk for inflammation and fibrosis. Because the common liver function tests, alanine aminotransferase (ALT) and aspartate aminotransferase (AST) lack sufficient sensitivity or specificity for NAFLD^20^,^21^,^22^,^23^,^24^ other non-invasive biomarkers have been studied, including those associated with cell death, inflammation, and oxidative stress, as well as algorithms of multi-component panels^25^. Thus far, no biomarkers have proven to be clinically acceptable for diagnosis, prognosis, or risk stratification^26^. Imaging techniques have also been used to assess NAFLD, including liver ultrasound, a relatively inexpensive modality with relatively high sensitivity for moderate to severe steatosis, magnetic resonance imaging (MRI) and magnetic resonance spectroscopy, both relatively expensive with limited availability, as well as computed tomography (CT) and transient ultrasound elastography^27^. However, none of the current imaging modalities are logistically or economically viable to broadly apply to the population at risk for NAFLD except as an adjunct to biopsy^28^.

We used a multivariate approach integrating “omics” derived variables from three data domains; genomic, phenomic, and proteomic. We selected the NAFLD susceptibility single nucleotide polymorphism (SNP) rs738409 in the patatin-like phospholipase domain containing 3 gene (PNPLA3)^29^ as the genomic variable, well characterized across diverse populations^30^,^31^,^32^. Phenomic variables were identified initially in a comprehensive analysis over 200 clinical variables^9^ and a set of serum proteins were identified using a novel unbiased high content multiplexed proteomic screen^33^ based on the SOMAmer^®^ (Slow Off-rate Modified Aptamer) technology. This approach uses single stranded DNA-based protein affinity reagents^21^ that incorporate chemically modified nucleotides as structural mimetics of amino acid side chains increasing diversity, affinity, and specificity for native proteins^22^. A multicomponent panel for steatosis using data from all three domains resulted in an AUC for the receiver operating characteristic (ROC) curve of 0.935. Integration of variables from a diverse set of data sources provides a powerful approach for the development of non-invasive biomarker algorithms for NAFLD.

## Materials and Methods

### Study Participants

Blood for DNA isolation and fasting serum samples were collected within three months prior to surgery during pre-operative clinic visits from 577 patients who had been consented as part of a research program on NAFLD and obesity in the Geisinger Clinic Center for Nutrition and Weight Management Bariatric Surgery Program. Study participants were randomly divided into a discovery (n = 443) or validation (n = 134) cohort. Intra-operative wedge biopsies of the liver and clinical data were obtained as previously described^23^. Patients with any evidence of hepatitis B virus (HBV), hepatitis C virus (HCV), or human immunodeficiency virus (HIV) infection, or alcohol abuse were included in this study as previously described^9^. A required comprehensive behavioral evaluation performed by a clinical psychologist included inquiries about current and past substance and alcohol use. If a patient used alcohol, they were further evaluated using established criteria for alcohol use disorders^24^. Patients whose clinical criteria for alcohol use disorders were denied access to bariatric surgery. In addition, patients with diagnosis codes ICD9 303 or ICD9 305.0 indicating a clinical diagnosis of alcohol abuse were also excluded. Source data included patient demographics, clinical measures, ICD9 codes, medical history, medication codes, and lab results. The research protocol was approved by the Geisinger Clinic Institutional Review Board, all participants provided written informed consent, and all experiments were performed in accordance with relevant guidelines and regulations.

### SOMAscan Assay

Serum samples were analyzed using the SOMAscan assay (SomaLogic; Boulder, CO), which is a sensitive, and quantitative protein biomarker discovery platform. SOMAmers (Slow Off-rate Modified Aptamers), single-stranded DNA aptamers with modified nucleotides, bind to specific proteins in the serum that are then be quantified as DNA. The SOMAscan assay quantified a total of 1129 proteins in each sample. In our analysis, the median lower limit of quantitation for all measured proteins was 0.3 picomolar (pM), with a dynamic range of >5 logs, and a median coefficient of variation (%CV) of 5%^34^.

### PNPLA3 genotyping

We extracted DNA from blood samples using standard methods^35^ and genotype marker rs738409 in the *PNPLA3* gene as described^36^.

### Phenomic modeling

Previously, we conducted univariate logistic regression to determine which of more than 200 pre-operative clinical variables were independently associated with the presence of steatosis in ~2300 individuals^9^. We identified 19 candidate variables associated with the presence of liver fat (Supplemental Table 1) and used logistic regression within the discovery cohort to identify the minimal subset of these variables that maintained the area under the curve (c-statistic) of the full model for steatosis. A backwards stepwise process was used for model variable selection. The initial model included all 19 variables. Subsequent models were evaluated by sequentially removing one variable at a time and assessing the resulting change in the c-statistic. When the removal of a variable resulted in a decrease in the c-statistic by <0.01, the variable was excluded from the final model. The variables retained for the final model were combined into a single classifier score by calculating (Supplemental Table S4) the predicted probability of steatosis. This score was applied to the validation cohort and brought forward for multi-component modeling.

### Derivation of Proteomic Panel

Candidate markers were selected using a stability selection algorithm with an L1-regularized logistic regression kernel. Stability selection takes many subsets of half the data and performs biomarker selection using the lasso classifier, which is a regularized logistic regression model^37^. The selection path for a single biomarker is the proportion of these subsets for which that biomarker was selected by the lasso model over a range of lambda, a tuning parameter that determines how many biomarkers are selected by the lasso. The maximum selection probability over a range of lambda values was the ultimate metric used to select a set of biomarkers.

Steatosis classifier models (steatosis vs. all other groups) were developed by inputting the most stable markers into the logistic regression classification algorithm. Once the models were fixed, bootstrap performance was done as verification of the developed models: the discovery set for steatosis as well as fibrosis were split randomly into 80% training and 20% test set to verify by bootstrapping. This was repeated 2500 times with a different subset. Sensitivity and specificity and confidence interval for each comparison was noted. The model types were evaluated using 10-fold cross-validation and inspecting plots of log-likelihood ratios and receiver operating characteristic (ROC) curves. The proteins retained for the final model were combined into a single classifier score by calculating the predicted probability of steatosis. This score was applied to the validation cohort and was brought forward for multi-component modeling.

### Multi-component modeling

The classifier scores from each domain were combined into logistic regression models. Performance of the combined classifiers was evaluated using area under the receiver operating characteristic (ROC) curve based on a c-statistic and 95% bootstrap confidence intervals.

## Results

### Characteristics of the discovery and validation cohorts

The study population of 576 adult patients was randomly assigned to either a discovery (N = 443) or validation (N = 134) cohort. As shown in Table 1, there were no significant differences in age, sex, body mass index (BMI), type 2 diabetes (T2D) status, measures of cholesterol metabolism, ALT and AST levels, platelet count, or *PNPLA3* genotype distribution between the two groups. Approximately 30% of the patients had normal liver histology (<5% steatosis), while 20–24% were classified as mildly or severely steatotic (5–33% and >66% liver fat, respectively) and ~25% showed moderate steatosis (33–66% fat). In both the discovery and validation cohorts, most of the key variables were different between patients with steatosis compared to those with normal liver histology (Supplemental Tables S1 and S2). For example, the percentage of patients with T2D increased from 22% in individuals with normal histology to 44% in those with steatosis, consistent with earlier findings reported by us^9^ and others^37^.

### Steatosis genomic classifier

For the genomic model, the *PNPLA3* rs738409 genotype was used as the sole variable, given the strength of its association with steatosis compared to other reported genetic variants^39^,^40^,^41^. Marker genotype was designated as homozygous wildtype, heterozygous, or homozygous NAFLD risk allele. As expected, the distribution of genotypes was significantly different between patients with steatosis compared to those with normal liver histology (Supplemental Tables S1 and S2).

### Steatosis phenomic classifier

We previously analyzed a cohort of 2929 subjects^9^ from which the 576 individuals used for the current analyses were drawn. Of the 19 variables previously identified (Supplemental Table S3), 12 were included in the final steatosis phenomic classifier including glucose, serum insulin, triglycerides, HDL, ALT, ferritin, creatinine, chloride, zinc, use of metformin, use of estrogen/progestin, and a clinical diagnosis of sleep apnea. These were combined into a single classifier score and used for multi-component modeling.

### Steatosis proteomic classifier

**Figure 1:**
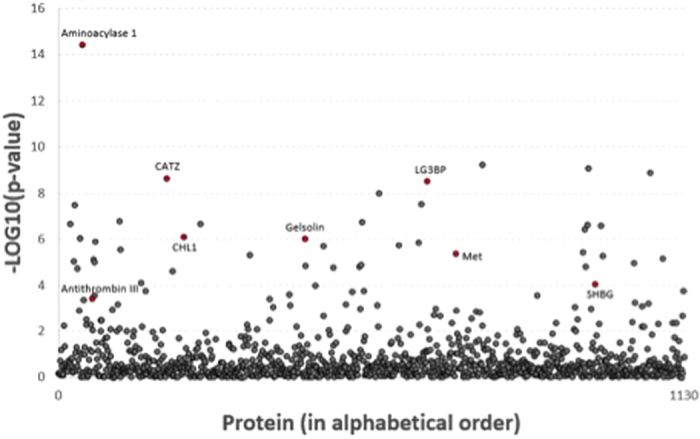
Plot of unadjusted p-values for association between each protein expression and presence of any steatosis. The labeled proteins were those selected for inclusion in final model.

Univariate analysis of serum levels of the SOMAmer platform of 1129 proteins using discovery and validation sets identified 30 proteins that met the Bonferroni-corrected level of statistical significance (Fig. 1). In multivariate analysis, eight proteins were associated with steatosis (Table 2). Serum levels of three of these proteins were associated with increased steatosis, while levels of five showed an inverse relationship with steatosis grade.

### Steatosis multi-component classifier

**Figure 2:**
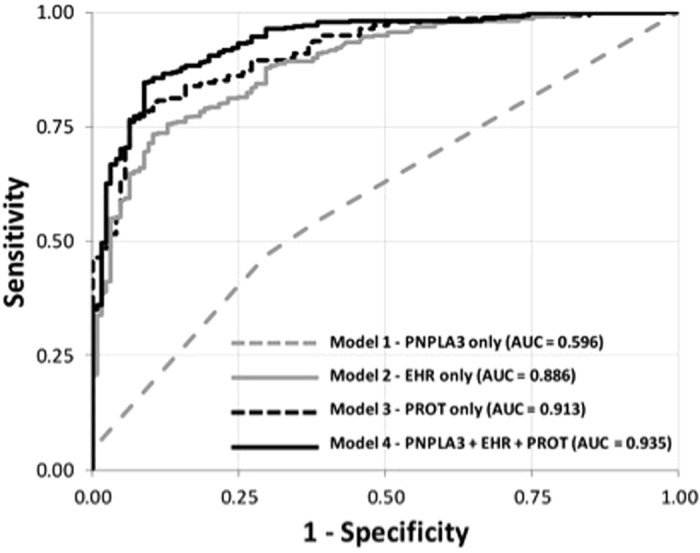
ROC curves for Discovery Model*. *Note that PNPLA3 was included as the number of alleles (0, 1, 2) and was treated as an ordinal variable. Those with unknown PNPLA3 status were included in the model by using a common missing data strategy (i.e. treating them as a separate subgroup).

**Figure 3:**
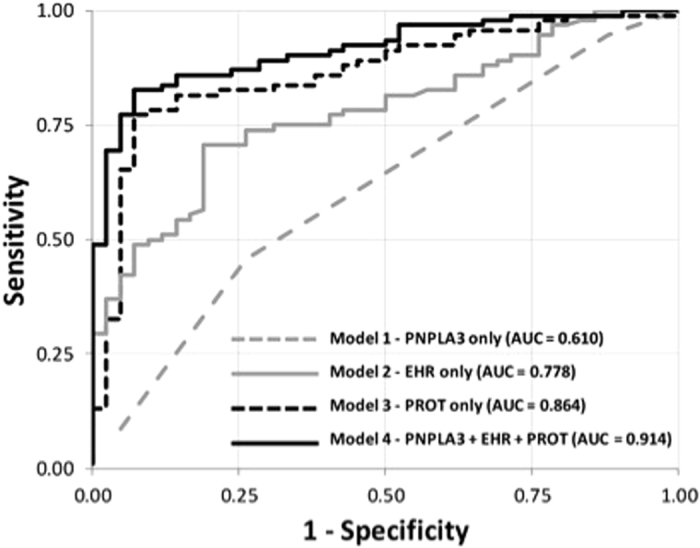
ROC curves for Validation model.

The results from modeling within each of the three individual data domains were then used to create a multi-component classifier that included *PNPLA3* genotype, steatosis clinical prediction score, and the proteomic classifier. We combined independently associated variables from each of the three data domains to generate a single logistic regression model and assessed performance of the combined classifier using area under (AUC) the receiver operating characteristic (ROC) curve based on a c-statistic (Table 3, Fig. 2). The proteomic classifier by itself achieved the highest AUC of the three individual data domains with an AUC of 0.913 versus an AUC for the *PNPLA3* genomic domain of only 0.596 and an AUC for the phenomic domain of 0.886. Combining genomic and phenomic domains yielded little effect on the AUC (0.892), while combining the phenomic and proteomic domains resulted in an AUC of 0.932. The highest AUC was achieved with the combination of all three domains, yielding a value of 0.935. A similar analysis was conducted using the validation cohort (Table 3, Fig. 3). Although the AUC values were lower across all validation models, the AUC values improved with the inclusion of each addition domain.

## Discussion

The central hallmark of NAFLD is the presence of increased fat in the liver, a condition that has several potentially important pathophysiological implications. For example, steatosis has been associated with the metabolic syndrome and insulin resistance, although the cause-effect relationship of this association is not clear^42^,^43^,^44^. Patients with steatosis are also at greater risk for the development of steatohepatitis and hepatic fibrosis, including cirrhosis^45^. The cause of NAFLD appears to be multifactorial^46^, although lifestyle (i.e., over-nutrition) plays a significant role by contributing to the development of obesity^47^. Both genetic variants^28^,^39^,^48^ and protein biomarkers^49^ have also been associated with NAFLD. Due to the underlying complexity of NAFLD pathogenesis, we sought to develop an algorithm based on the unbiased assessment of large groups of variables, i.e., using “omics” approaches, in several data domains that could differentiate NAFLD in a population with extreme obesity. A similar type of “omics” approach combining transcriptomic, ELISA-based serum proteomic, and nuclear magnetic resonance-based metabolomic analyses of liver biopsy tissue and serum samples obtained from patients with high versus low grade steatosis has recently been reported^50^. However, the number of individuals assessed was quite small (N = 20), thereby limiting the conclusions that can be drawn from that analysis.

We selected the rs738409 variant in *PNPLA3* for modeling based on existing genomic data that had been generated in populations not selected for obesity^39^,^40^,^41^, as well as our own data based on a similar population with extreme obesity^39^. By itself, rs738409 showed the poorest discriminatory value, a finding that was not surprising given the relatively small effect size found in the initial studies^51^,^52^. Adding rs738409 genotype to the phenomic classifier essentially yielded no effect, whereas combining it with the proteomic classifier had a small but positive effect (0.892 vs. 0.913). This suggests that genomic classifier may be already represented by one or more variables present in the phenomic classifier. This is somewhat surprising because the phenomic variables are largely represented by those related to metabolic abnormalities, while rs738409 genotype has been associated with NAFLD independent of metabolic disease^48^,^53^,^54^, although some studies have found an interaction with glucose metabolism^55^. Its complementary relationship with the group of serum proteins in the proteomic classifier implies that there are multiple mechanistic pathways involved in the development of steatosis.

We used the SOMAscan platform^21^,^32^,^56^ as a discovery assay that has been applied successfully to diagnostic biomarker discovery and validation for other disorders. This platform has been used for the identification of biomarkers in rheumatoid arthritis^57^, Alzheimer’s disease^58^, and infectious disease^59^. The SOMAscan aptamer-based assay has been designed for high-throughput multiplexing allowing for the measurement of over 1000 proteins in only 65 μL of serum. However, because the aptamer-based methodology only detects available protein epitopes, in instances where epitopes may be blocked by other proteins or post-translational modifications, measured levels may not represent actual protein concentrations. Further, protein markers associated with steatosis were identified using a stability selection algorithm with an L1-regularized logistic regression kernel; therefore, the most stable group of markers may not represent the most highly associated individual markers. Nevertheless, individual markers identified here may shed light on the underlying biology of steatosis. For example, several of the serum proteins identified in the proteomic screen have previously been associated with NAFLD or a related aspect of hepatic lipid metabolism. Aminoacylase 1, a zinc-binding protein that catalyzes the hydrolysis of N-acetyl amino acids into free aliphatic amino acids and acetic acid^60^, was increased in hepatic lipid droplets of mice subjected to caloric restriction^61^, suggesting a role in the metabolic adjustments to the overfed state in NAFLD. However, we found increased steatosis associated with aminoacylase 1 levels. This could reflect a rapid temporal response in aminoacylase 1 levels since patients were fasting at the time of blood draw. In addition, sex hormone binding globulin (SHBG), a glycoprotein that is produced primarily by hepatocytes and serves to transport sex steroid hormones through the blood to target tissues, was first associated with hepatic steatosis through studies of monosaccharide-induced hepatic lipogenesis in animals, a treatment that suppressed expression of sex hormone–binding globulin^62^. A number of human population-based studies have found that SHBG levels are inversely associated with NAFLD^63^,^64^,^65^,^66^,^67^,^68^, consistent with our results. MET (hepatocyte growth factor)/mesenchymal-epithelial transition factor) functions in anti-apoptosis pathway signaling in hepatocytes, in part by sequestering Fas to inhibit Fas-mediated apoptosis. This relationship appears to be lost in NAFLD^69^.

Data linking the other protein markers to NAFLD is less clear. Galectin-3 binding protein (LGALS3BP) has been used as a component of a multi-protein panel for the prediction of fibrosis in Hepatitis C (HCV) infection^70^, and serves as a biomarker of hepatocellular carcinoma resulting from HCV cirrhosis^71^. Antithrombin III levels did not correlate with liver NAFLD histology in obese patients^72^, although markedly decreased levels have been found in acute fatty liver of pregnancy^73^. Gelsolin is a protein generated by the liver, and appears to be expressed by hepatic sinusoidal endothelial cells, hepatic stellate cells, myofibroblasts, and mononuclear cells, but not hepatocytes^74^. Gelsolin has been implicated in the apoptosis of hepatic stellate cells, which play a major role in the progression of steatosis to fibrosis and cirrhosis^75^,^76^. Deficiency of cell adhesion molecule L1 like does not appear to affect hepatic metabolism. Chl1 knockout mice did not show any significant abnormal phenotype up to an age of 2 years^77^. Little biological information is available on cathepsin Z, a cysteine proteinase of the papain family, except that it is widely expressed in human tissues^78^.

In addition to non-invasive blood-based classifiers, various imaging approaches have been used to characterize NAFLD. Ultrasound is commonly used to assess for the presence and amount of liver fat, though it can be dependent upon the skill of the operator and the particular capacity of the instrument^79^. Due to the subjective nature of the technique, reproducibility can therefore be low and the ability to detect mild levels of steatosis is not as robust as for moderate to severe steatosis. However, the technique is relatively inexpensive, simple to perform, and safe. Alternatives to traditional ultrasound, such as controlled attenuation parameter^80^, are being developed and validated, thus may significantly improve on the disadvantages. Other imaging modalities include computed tomography (CT) and magnetic resonance imaging (MRI). Multi-parametric quantitative MRI is also a promising technique that may be able to closely correlate with liver histology. However, all imaging-based approaches are limited by cost and availability vis-à-vis blood-based testing and will likely not be scalable to the population at risk for NAFLD.

We used variables from three different data domains that were derived from “omics” analyses to develop a multi-component classifier for NAFLD. Despite the robust nature of the classifier, there is still a need to improve the AUC. The use of even larger samples sizes would be useful, although high-throughput analyses are more costly and complex than smaller scale analyses. We also have used a population with extreme levels of obesity in part because of the availability of gold standard liver biopsy pathology data. Developing classifiers for lower levels of obesity may be limited by the difficulties in obtaining such data. We also used a relatively ethnically, racially, and geographically homogenous population, appropriate for discovery and initial development studies. Extending these results to other populations and/or developing classifiers specific to other populations are needed for future studies. Despite these potential limitations, our results suggest that a high-throughput, multi-domain, multi-component approach may be a promising avenue for further investigation.

## Additional Information

**How to cite this article**: Wood, G. C. *et al*. A multi-component classifier for nonalcoholic fatty liver disease (NAFLD) based on genomic, proteomic, and phenomic data domains. *Sci. Rep.* **7**, 43238; doi: 10.1038/srep43238 (2017).

**Publisher's note:** Springer Nature remains neutral with regard to jurisdictional claims in published maps and institutional affiliations.

## Supplementary Material

Supplementary Information

## Figures and Tables

**Table 1:** Characteristics of discovery and validation sets.

Variable	Measure	Discovery group N = 443	Validation group N = 134	p-value
Age, years	Mean (SD)	46.2 (10.7)	46.3 (11.1)	0.943^1^
Sex	Female, % (n)	82% (n = 363)	84% (n = 112)	0.663^2^
	Male, % (n)	18% (n = 80)	16% (n = 22)	
Race	White, % (n)	99% (n = 439)	99% (n = 133)	0.999^3^
	Black, % (n)	< 1% (n = 2)	1% (n = 1)	
	Other, % (n)	<1% (n = 2)	0% (n = 0)	
BMI, kg/m^2^	Mean (SD)	49.2 (9.0)	49.2 (8.4)	0.979^1^
Diabetes	Yes, % (n)	41% (n = 180)	41% (n = 55)	0.932^2^
Hypertension	Yes, % (n)	47% (n = 209)	44% (n = 59)	0.522^2^
Dyslipidemia	Yes, % (n)	37% (n = 163)	43% (n = 57)	0.230^2^
ALT, U/L	Median [IQR]	27 [20, 39]	26 [19, 38]	0.498^4^
AST, U/L	Median [IQR]	24 [19, 33]	24 [20, 30]	0.647^4^
Cholesterol, md/dL	Mean (SD)	187.7 (40.3)	188.2 (39.5)	0.897^1^
HDL, md/dL	Mean (SD)	47.2 (11.5)	46.2 (10.8)	0.351^1^
LDL, md/dL	Mean (SD)	105.8 (33.6)	107.4 (36.1)	0.630^1^
Triglycerides, md/dL	Median [IQR]	152 [104, 208]	180.6 (118.9)	0.911^4^
Platelet count, K/uL	Mean (SD)	285.2 (72.3)	294.7 (64.2)	0.175^1^
Steatosis	<5%, % (n)	30% (n = 131)	32% (n = 43)	0.612^2^
	5–33%, % (n)	20% (n = 89)	24% (n = 32)	
	33–66%, % (n)	26% (n = 117)	24% (n = 32)	
	>66%, % (n)	24% (n = 106)	20% (n = 27)	
Lobular inflammation	No foci, % (n)	55% (n = 242)	62% (n = 83)	0.157^2^
	<2 foci*, % (n)	37% (n = 162)	34% (n = 45)	
	2–4 foci*, % (n)	9% (n = 39)	4% (n = 6)	
	>4 foci*, % (n)	0% (n = 0)	0% (n = 0)	
Fibrosis stage	None, % (n)	59% (n = 262)	64% (n = 86)	0.069^3^
	1, % (n)	25% (n = 111)	28% (n = 37)	
	2, % (n)	9% (n = 39)	7% (n = 10)	
	3, % (n)	5% (n = 20)	1% (n = 1)	
	4, % (n)	2% (n = 11)	0% (n = 0)	
PNPLA3**	CC, % (n)	54% (n = 219)	57% (n = 71)	0.407^2^
	CG, % (n)	40% (n = 163)	35% (n = 43)	
	GG, % (n)	6% (n = 23)	8% (n = 10)	

Reference ranges: ALT (Male 5–52 U/L, Female 10–60 U/L), AST (Male 13–39 U/L, Female 10–42 U/L), Cholesterol (<200 mg/dL), HDL (> = 40 mg/dL), LDL (<130 mg/dL), Triglycerides (<150 mg/dL), Platelet Count (140–400 K/uL).

^1^Two-sample t-test; ^2^Chi-square test; ^3^Fisher’s Exact Test; ^4^Wilcoxon Rank-Sum test.

SD = standard deviation, IQR = Interquartile Range.

*per 200X field.

**PNPLA3 unknown for 48 patients (38 in discovery group and 10 in the validation group). Hardy-Weinberg test for equilibrium: p = 0.304 in discovery group and p = 0.344 in validation group.

**Table 2:** Logistic regression model for NAFLD using selected protein biomarkers.

Gene	Protein	Odds Ratio	[95% CI]	p-value
ACY1	Aminoacylase-1	57.89	[13.69, 244.90]	<0.0001
SHBG	Sex hormone-binding globulin	0.56	[0.42, 0.75]	<0.0001
CTSZ	Cathepsin Z	0.69	[0.48, 0.98]	0.0400
MET	Hepatocyte growth factor receptor	0.60	[0.43, 0.83]	0.0020
GSN	Gelsolin/GSN	2.69	[1.74, 4.16]	<0.0001
LGALS3BP	Galectin-3 binding protein	0.59	[0.43, 0.79]	0.0005
CHL1	Neural cell adhesion molecule L1-like protein	2.20	[1.42, 3.42]	0.0004
SERPINC1	Antithrombin III	0.68	[0.49, 0.94]	0.0185

The biomarkers were rescaled to the standard normal (mean = 0, SD = 1) before inclusion in the logistic regression model. Odds ratios can be interpreted as the odds of steatosis for each 1 standard deviation increase in the protein expression level.

**Table 3:** Area under (AUC) the receiver operating characteristic (ROC) curve based on a c-statistic.

Model	Discovery		Validation	
	AUC	95%CI	AUC	95%CI
1. GENOMIC only	0.596	[0.547, 0.645]	0.610	[0.519, 0.713]
2. PHENOMIC only	0.886	[0.851, 0.918]	0.778	[0.693, 0.851]
3. PHENO + GENO	0.892	[0.862, 0.924]	0.782	[0.710, 0.865]
4. PROTEOMIC ONLY	0.913	[0.882, 0.937]	0.864	[0.793, 0.927]
5. PROTEO + GENO	0.920	[0.892, 0.946]	0.889	[0.832, 0.945]
6. PROTEO + PHENO	0.932	[0.904, 0.955]	0.892	[0.840, 0.943]
7. 3 DOMAIN MODEL	0.935	[0.913, 0.959]	0.914	[0.871, 0.957]
