# Supplementary material for: A multi-component classifier for nonalcoholic fatty liver disease (NAFLD) based on genomic, proteomic, and phenomic data domains
Source: Sci Rep. 2017 Mar 7;7:43238. doi: 10.1038/srep43238 (PMC5339694; doi:10.1038/srep43238)
Supplement: Supplementary Information [file srep43238-s1.doc]

Supplementary Information

**A multi-component classifier for nonalcoholic fatty liver disease (NAFLD) based on genomic, proteomic, and phenomic data domains.**

G. Craig Wood, Xin Chu, George Argyropoulos, Peter Benotti, David Rolston, Tooraj Mirshahi, Anthony Petrick, John Gabrielson, David J. Carey, Johanna K. DiStefano, Christopher D. Still, and Glenn S. Gerhard

1. Table S1. Patient characteristics compared between steatosis and no steatosis in the Discovery Group.

2. Table S2. Patient characteristics compared between steatosis and no steatosis in the Validation Group.

3. Table S3. Multiple regression results for presence of steatosis.

4. Calculation of phenomic classifier.

**Table S1. Patient characteristics compared between steatosis and no steatosis** in the Discovery Group.

|  |  | **Any Steatosis**  **N=318** | **No steatosis**  **N=125** | **p-value** |
| --- | --- | --- | --- | --- |
| Age, years | Mean (SD) | 46.9 (10.2) | 44.6 (11.7) | 0.042^1^ |
| Sex | Female, % (n) | 79% (n=252) | 89% (n=111) | 0.019^2^ |
|  | Male, % (n) | 21% (n=66) | 11% (n=14) |  |
| Race | White, % (n) | 99% (n=314) | 100% (n=125) | 0.999^3^ |
|  | Black, % (n) | <1% (n=2) | 0% (n=0) |  |
|  | Other, % (n) | <1% (n=2) | 0% (n=0) |  |
| BMI, kg/m^2^ | Mean (SD) | 49.0 (8.3) | 49.7 (10.4) | 0.481^1^ |
| Diabetes | Yes, % (n) | 48% (n=152) | 22% (n=28) | <0.0001^2^ |
| Hypertension | Yes, % (n) | 52% (n=164) | 36% (n=45) | 0.0031^2^ |
| Dyslipidemia | Yes, % (n) | 40% (n=127) | 29% (n=36) | 0.029^2^ |
| ALT, U/L | Median [IQR] | 30 (22, 44] | 20 [16, 26] | <0.0001^4^ |
| AST, U/L | Median [IQR] | 27 [21, 35] | 21 [17, 24] | <0.0001^4^ |
| Cholesterol, md/dL | Mean (SD) | 188.5 (41.6) | 185.7 (36.8) | 0.518^1^ |
| HDL, md/dL | Mean (SD) | 45.7 (10.6) | 51.1 (12.7) | <0.0001^1^ |
| LDL, md/dL | Mean (SD) | 104.9 (33.9) | 107.9 (33.0) | 0.411^1^ |
| Triglycerides, md/dL | Median [IQR] | 167 [118, 231] | 117 [85, 168] | <0.0001^4^ |
| Platelet count, K/uL | Mean (SD) | 280.7 (68.4) | 296.8 (30.4) | 0.036^1^ |
| PNPLA3* | CC, % (n) | 49% (n=143) | 67% (n=76) | 0.0016^3^ |
|  | CG, % (n) | 44% (n=128) | 31% (n=35) |  |
|  | GG, % (n) | 7% (n=21) | 2% (n=2) |  |

Normal ranges for the lab values were ALT (Male 5-52 U/L, Female 10-60 U/L), AST (Male 13-39 U/L, Female 10-42 U/L), total cholesterol (<200 mg/dL), HDL (>=40 mg/dL), LDL (<130 mg/dL), triglycerides (<150 mg/dL), platelet count (140-400 K/uL)

^1^Two-sample t-test; ^2^Chi-square test; ^3^Fisher’s Exact Test; ^3^Wilcoxon Rank-Sum test

SD=standard deviation, IQR=Interquartile Range

*PNPLA3 unknown for 38 patients (26 in steatosis group and 12 in the no steatosis group).

**Table S2. Patient characteristics compared between steatosis and no steatosis** in the Validation Group.

|  |  | **Any Steatosis**  **N=92** | **No steatosis**  **N=42** | **p-value** |
| --- | --- | --- | --- | --- |
| Age, years | Mean (SD) | 46.8 (10.6) | 45.3 (12.4) | 0.493^1^ |
| Sex | Female, % (n) | 82% (n=75) | 88% (n=37) | 0.341^2^ |
|  | Male, % (n) | 18% (n=17) | 12% (n=5) |  |
| Race | White, % (n) | 99% (n=91) | 100% (n=42) | 0.999^3^ |
|  | Black, % (n) | 1% (n=1) | 0% (n=0) |  |
|  | Other, % (n) | 0% (n=0) | 0% (n=0) |  |
| BMI, kg/m^2^ | Mean (SD) | 49.1 (9.0) | 49.5 (7.0) | 0.800^1^ |
| Diabetes | Yes, % (n) | 46% (n=42) | 31% (n=13) | 0.109^2^ |
| Hypertension | Yes, % (n) | 49% (n=45) | 33% (n=14) | 0.092^2^ |
| Dyslipidemia | Yes, % (n) | 40% (n=37) | 48% (n=20) | 0.421^2^ |
| ALT, U/L | Median [IQR] | 30 [21, 41] | 22 [16, 26] | <0.0001^4^ |
| AST, U/L | Median [IQR] | 25 [20, 31] | 23 [18, 27] | 0.024^4^ |
| Cholesterol, md/dL | Mean (SD) | 187.0 (39.0) | 190.7 (40.8) | 0.615^1^ |
| HDL, md/dL | Mean (SD) | 44.7 (10.3) | 49.3 (11.3) | 0.023^1^ |
| LDL, md/dL | Mean (SD) | 104.8 (34.7) | 113.0 (38.8) | 0.221^1^ |
| Triglycerides, md/dL | Median [IQR] | 156 [119, 243] | 136 [103, 169] | 0.012^4^ |
| Platelet count, K/uL | Mean (SD) | 295.8 (69.2) | 292.3 (52.2) | 0.770^1^ |
| PNPLA3* | CC, % (n) | 52% (n=45) | 70% (n=26) | 0.173^3^ |
|  | CG, % (n) | 39% (n=34) | 24% (n=9) |  |
|  | GG, % (n) | 9% (n=8) | 5% (n=2) |  |

Normal ranges for the lab values were ALT (Male 5-52 U/L, Female 10-60 U/L), AST (Male 13-39 U/L, Female 10-42 U/L), total cholesterol (<200 mg/dL), HDL (>=40 mg/dL), LDL (<130 mg/dL), triglycerides (<150 mg/dL), platelet count (140-400 K/uL)

^1^Two-sample t-test; ^2^Chi-square test; ^3^Fisher’s Exact Test; ^3^Wilcoxon Rank-Sum test

SD=standard deviation, IQR=Interquartile Range

*PNPLA3 unknown for 10 patients (5 in steatosis group and 5 in the no steatosis group).

**Table S3**. Multiple regression results for presence of steatosis.

|  | **Odds ratio** | **95% Confidence interval** | **p-value** |
| --- | --- | --- | --- |
| **Glucose** level: <100 mg/dL | Reference |  |  |
| 100-124 mg/dL | 1.86 | [0.82, 4.24] | 0.139 |
| 125-199 mg/dL | 3.48 | [1.08, 11.18] | 0.036 |
| 200+ mg/dL | 2.39 | [0.49, 11.76] | 0.282 |
| Serum **insulin** >17 µU/ml | 4.50 | [2.47, 8.19] | <0.0001 |
| **Triglyceride** level: <125 mg/dL | Reference |  |  |
| 125-199 mg/dL | 1.39 | [0.74, 2.60] | 0.301 |
| 200+ mg/dL | 3.82 | [1.62, 9.00] | 0.0022 |
| **HDL** <50 mg/dL | 1.82 | [1.02, 3.25] | 0.044 |
| **ALT** level: Quartile 1 (<20 U/L) | Reference |  |  |
| Quartile 2: (20-25 U/L) | 1.61 | [0.79, 3.29] | 0.192 |
| Quartile 3: (26-36 U/L) | 5.39 | [2.39, 12.13] | <0.0001 |
| Quartile 4: (37+ U/L) | 4.60 | [2.09, 10.11] | 0.0001 |
| Elevated **ferritin** (>400 ng/mL M, >100 ng/mL F) | 2.57 | [1.15, 5.74] | 0.021 |
| **Creatinine** level: Low/Normal (<1.1 mg/dL) | Reference |  |  |
| High (1.2+ mg/dL) | 0.09 | [0.03, 0.28] | <0.0001 |
| **Chloride** level: Quartile 1 or 2 (<103 mmol/L) | 1.64 | [0.94, 2.85] | 0.080 |
| Quartile 3 or 4 (103+ mmol/L) | Reference |  |  |
| Use of **metformin** | 3.10 | [1.69, 5.70] | 0.0003 |
| Use of **Estrogen/Progestin** | 0.30 | [0.12, 0.74] | 0.0090 |
| **Sleep apnea** diagnosis | 2.25 | [1.24, 4.09] | 0.0078 |

c-statistic=0.886

**Calculation of phenomic classifier**. The parameter estimates from the logistic regression model were used to calculate the predicted probability of steatosis (which was used as the phenomic classifier). The steps in the calculation were:

Calculate log odds: LOGODDS = -2.4953 + (0.6216*GLUCOSE1) + (1.2462* GLUCOSE2) + (0.873* GLUCOSE3) + (1.5043*INSULIN) + (0.3297*TRIG1) + (1.3388*TRIG2) + (0.5966*HDL) + (0.4761*ALT1) + (1.6842*ALT2) + (1.526*ALT3) + (0.9453*FERRITIN) + (-2.3768*CREAT) + (0.4945*CHLORIDE) + (1.1319*METFORMIN) + (-1.2182*ESTROGEN) + (0.8102*SLEEP), where

- GLUCOSE1=1 if glucose 100-124 mg/dL, otherwise=0
- GLUCOSE2=1 if glucose 125-199 mg/dL, otherwise=0
- GLUCOSE3=1 if glucose 200+ mg/dL, otherwise=0
- INSULIN=1 if insulin >17 µU/ml, otherwise=0
- TRIG1=1 if triglycerides 125-199 mg/dL, otherwise=0
- TRIG2=1 if triglycerides 200+ mg/dL, otherwise=0
- HLD=1 if HDL <50 mg/dL, otherwise=0
- ALT1=1 if ALT 20-25 U/L, otherwise=0
- ALT2=1 if ALT 26-36 U/L, otherwise=0
- ALT3=1 if ALT 37+ U/L, otherwise=0
- FERRITIN=1 if ferritin >400 ng/mL M, >100 ng/mL F, otherwise=0
- CREAT=1 if creatinine 1.2+ mg/dL, otherwise=0
- CHLORIDE=1 if chloride <103 mmol/L, otherwise=0
- METFORMIN=if using metformin, otherwise=0
- ESTROGEN=if using estrogen or progestin, otherwise=0
- SLEEP=1 if there is a diagnosis of sleep apnea, otherwise=0

Convert log odds to odds. ODDS = exp(LOGODDS)

Convert to predicted probability. PROB = ODDS / (1 + ODDS).
